# DrugEx v3: scaffold-constrained drug design with graph transformer-based reinforcement learning

**DOI:** 10.1186/s13321-023-00694-z

**Published:** 2023-02-20

**Authors:** Xuhan Liu, Kai Ye, Herman W. T. van Vlijmen, Adriaan P. IJzerman, Gerard J. P. van Westen

**Affiliations:** 1 Drug Discovery and Safety, Leiden Academic Centre for Drug Research, Einsteinweg 55, Leiden, The Netherlands; 2 School of Electrics and Information Engineering, Xi’an Jiaotong University, 28 XianningW Rd, Xi’an, China; 3 Janssen Pharmaceutica NV, Turnhoutseweg 30, B-2340 Beerse, Belgium

**Keywords:** Deep learning, Reinforcement learning, Policy gradient, Drug design, Transformer, Multi-objective optimization, Adenosine A_2A_ receptor

## Abstract

Rational drug design often starts from specific scaffolds to which side chains/substituents are added or modified due to the large drug-like chemical space available to search for novel drug-like molecules. With the rapid growth of deep learning in drug discovery, a variety of effective approaches have been developed for de novo drug design. In previous work we proposed a method named *DrugEx*, which can be applied in polypharmacology based on multi-objective deep reinforcement learning. However, the previous version is trained under fixed objectives and does not allow users to input any prior information (*i.e.* a desired scaffold). In order to improve the general applicability, we updated *DrugEx* to design drug molecules based on scaffolds which consist of multiple fragments provided by users. Here, a  Transformer model was employed to generate molecular structures. The Transformer is a multi-head self-attention deep learning model containing an encoder to receive scaffolds as input and a decoder to generate molecules as output. In order to deal with the graph representation of molecules a novel positional encoding for each atom and bond based on an adjacency matrix was proposed, extending the architecture of the Transformer. The graph Transformer model contains growing and connecting procedures for molecule generation starting from  a given scaffold based on fragments. Moreover, the generator was trained under a reinforcement learning framework to increase the number of desired ligands. As a proof of concept, the method was applied to design ligands for the adenosine A_2A_ receptor (A_2A_AR) and compared with SMILES-based methods. The results show that 100% of the generated molecules are valid and most of them had a high predicted affinity value towards A_2A_AR with given scaffolds.

**Supplementary Information:**

The online version contains supplementary material available at 10.1186/s13321-023-00694-z.

## Introduction

Due to the size of drug-like chemical space (*i.e.* estimated at 10^33^–10^60^ organic molecules) [1] it is impossible to screen every corner of it to discover optimal drug candidates. Commonly, specific scaffolds derived from endogenous substances, high throughput screening, or a phenotypic assay [2] are taken as a starting point to design analogs while side chains/substituents are added or modified [3]. These fragments are used as “building blocks” to develop drug leads with e.g. combinatorial chemistry such as growing, linking, and merging [4]. After a promising drug lead has been discovered it is further optimized by modifying side chains to improve potency towards the relevant targets, to improve selectivity over off-targets, and physicochemical properties which in turn can improve safety and tolerability [5].

In scaffold-based rational drug design it is generally accepted that a chemical space consisting of 10^9^ diverse molecules can be sampled with only 10^3^ fragments [6]. For instance, one well known class of drug targets are G Protein-coupled receptors (GPCRs), a family via which approximately 35% of drug exert their effect [7]. The adenosine receptors (ARs) form a family within rhodopsin-like GPCRs and include four subtypes (A_1_, A_2A_, A_2B_ and A_3_). Each of them has a unique pharmacological profile, tissue distribution, and effector coupling [8, 9]. ARs are ubiquitously distributed throughout the human tissues, and involved in many biological processes and diseases [10]. As adenosine is the endogenous agonist of the ARs, a number of known ligands of the ARs are adenosine analogs and/or have a common scaffold. Examples of the latter include purines, xanthines, triazines, pyrimidines [11]. In this work, we aim to design novel ligands for this family of receptors with deep generative neural networks.

Deep learning methods have been gaining ground over the last decade in computational drug discovery, including de novo design [12]. Deep learning has achieved breakthroughs in visual recognition, natural language processing, and other data-rich fields [13]. In drug discovery the following developments rapidly followed each other. For distribution-directed issues, Gomez-Bombarelli et al*.* implemented variational autoencoders (VAE) to map molecules into a latent space where each point can also be decoded into unique molecules inversely [14]. They used recurrent neural networks (RNNs) to successfully learn SMILES (simplified molecular-input line-entry system) grammar and construct a distribution of molecular libraries [15]. For goal-directed issues, Sanchez-Lengeling et al*.* combined reinforcement learning and generative adversarial networks (GANs) to develop an approach named *ORGANIC* to design active compounds for a given target [16]. Olivecrona et al*.* proposed the *REINVENT* algorithm which updated this reinforcement learning with a Bayesian approach and combined RNNs to generate SMILES-based desired molecules [17, 18]. Moreover, Lim et al*.* proposed a method for scaffold-based molecular design with a graph generative model [19]. Li et al. also used deep learning to develop a tool named *DeepScaffold* for this issue [20]. Arús‑Pous et al*.* employed RNNs to develop a SMILES-based scaffold decorator for de novo drug design [21]. Finally, Yang et al*.* used the Transformer model [22] to develop a tool named *SyntaLinker* for automatic fragment linking [23]. Here we continue to address this issue further with different molecular representations and deep learning architectures.

In previous studies we investigated the performance of RNNs and proposed a method named *DrugEx* that balances distribution-directed and goal-directed tasks in reinforcement learning [24]. Subsequently, *DrugEx* was updated with multi-objective reinforcement learning and applied in a polypharmacology use case [25]. However, these models cannot receive any input data from users and can only produce a distribution of desired molecules with fixed conditions. If the objectives are changed, the model needs to be trained again. Here, different end-to-end deep learning methods are compared to update the *DrugEx* model to allow users to provide prior information, *e.g.* fragments that should occur in the generated molecules. Based on the extensive experience in our group with the A_2A_AR, this target is again used as an example to evaluate the performance of these novel methods. The Transformer model takes scaffolds composed of multiple fragments as input to generate desired molecules which are predicted to be active on A_2A_AR enabling scaffold-constrained drug design. All python code developed in this study is freely available at https://github.com/CDDLeiden/DrugEx.

## Materials and methods

### Data source

The *ChEMBL* set from *DrugEx v2* was reused [25]. This set consisted of small molecule compounds downloaded from ChEMBL using a SMILES notation (version 27) [26]. After data preprocessing via RDKit ~ 1.7 million molecules remained for model pre-training. Preprocessing included neutralizing charges and removing metals and small fragments. In addition, 10,828 ligands and their bioactivity data on one or more of the four human adenosine receptors were extracted from ChEMBL to construct the *LIGAND* set. The *LIGAND* set structures were used for fine-tuning the generative model. Moreover, molecules with annotated A_2A_AR activity were used to train a bioactivity prediction model. If multiple measurements for the same ligand existed, the average pChEMBL [27] value (pX, including pKi, pKd, pIC50 or pEC50) was calculated and duplicate items were removed. For the bioactivity models the threshold of affinity was defined as pX = 6.5 to predict if the compound was active (> = 6.5) or inactive (< 6.5). It was shown previously that this enables the creation of a balanced classifier [28].

**Fig. 1:**
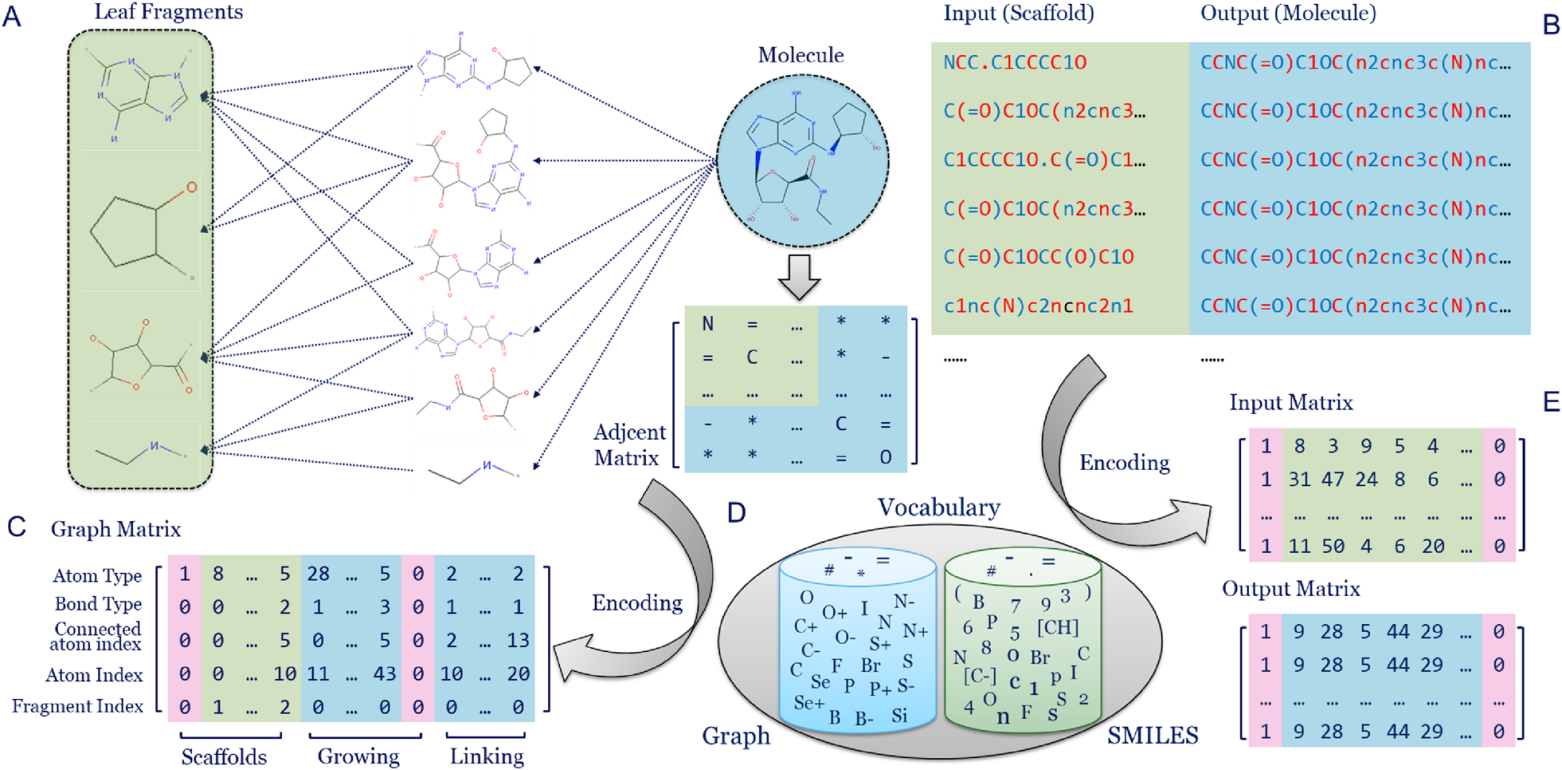
scaffold-molecule pair dataset construction. (A) Each molecule in the dataset is decomposed hierarchically into a series of fragments with the BRICS algorithm. (B) Subsequently data pairs between input and output are created. Combinations of leaf fragments form the scaffold as input, while the whole molecule becomes the output. For clarity token colors alternate. (C) After conversion to an adjacency matrix, each molecule was represented as a graph matrix. The graph matrix contains five rows, standing for the atom type, bond type, connected atom index, atom index, and fragment index. Columns are divided in three parts to store the information of the fragment, the growing section and the linking section. (D) All tokens are collected to construct the vocabularies for SMILES-based and graph-based generators, respectively. (E) An example of the input and output matrices for the SMILES representation of scaffolds and molecules.

The dataset was constructed with an input–output pair for each data point. Each molecule was decomposed into a series of fragments with the BRICS method [29] in RDKit (Fig. 1A). If a molecule contained more than four leaf fragments, the smaller fragments were ignored and a maximum of four larger fragments were kept. For example, if one molecule M contained four fragments, including A, B, C and D, then there were 15 input–output pairs (A-M, B-M, C-M, D-M, AB-M, AC-M, AD-M, BC-M, BD-M, CD-M, ABC-M, ABD-M, ACD-M, BCD-M, ABCD-M) based on the full combination. The order of input fragments was randomly selected. The SMILES of the fragments were joined with ‘.’ as input data and a pair was created with the full SMILES of molecules. The scaffold was defined as the combination of different fragments which can be either continuous (linked) or discrete (separated). The resulting scaffold-molecule pairs formed the input and output data (Fig. 1B). After completion of the data pairs, the set was split into a training set, a validation set, and a test set with the ratio 8:1:1 based on the scaffolds. As a result, the *ChEMBL* set contained 9,335,410 pairs in the training set, 1,104,125 pairs in the validation set, and 1,083,271 pairs in the test set. In addition, in the *LIGAND* set there were 53,888 pairs in the training set, 7,380 pairs in the validation set, and 7,525 pairs in the test set. Moreover, the scaffolds in *LIGAND* set were also split into training set (11,836 samples), validation set (1,479 samples), and test set (1,479 samples) with the ratio 8:1:1 for reinforcement learning.

## Molecular representations

Two different molecular representations were tested: SMILES and graph. For SMILES representations each scaffold-molecule pair was transformed into two SMILES sequences which were then split into different tokens to denote atoms, bonds, or tokens for grammar control (e.g. parentheses or numbers). All of these tokens were put together to form a vocabulary which recorded the index of each token (Fig. 1D). The same conversion procedure and vocabulary as in *DrugEx* v2 was used [25]. Summarizing, for both input and output sequences of each pair, a start token (GO) was put at the beginning and an end token (END) at the end. After sequence padding with a blank token at empty positions, each SMILES sequence was rewritten as a series of token indices with a fixed length to form the input and output matrix (Fig. 1E).

For the graph representation each molecule was represented as a five-row matrix, in which the first two rows stand for the atom type and and bond types, respectively. The third and fourth rows represent the connected atom index and current atom index, and the fifth row represents the fragment index (Fig. 1C). The columns of this matrix contain three sections to store the fragment, growing part, and linking part. The fragment section starts with a start token in the first row and the last row was labeled with the index of each fragment starting from one. The fragments of each molecule are put in the beginning of the matrix, followed by the growing part for the fragment, and the last part is the connecting bond between these growing fragments with single bonds. For the growing and linking sections the last row was always zero and these two sections were separated by the column of the end token. It is worth noticing that the last row was not directly involved in the training process. The vocabulary for graph representation was different from the SMILES representation, containing 38 atom types (Additional file 1: Table S1), and four bond types (single, double, triple bonds and no bond). For each column, if an atom is the first occurrence in a given fragment the type of the bond will be empty (indexed as 0 with token ‘*’). In addition, if the atom at the current position has occurred in the matrix, the type of the atom in this column will be empty. In order to grasp more details of the graph representation, the pseudocode for encoding (Additional file 1: Table S2) and decoding (Additional file 1: Table S3) is provided.

### End-to-end deep learning

**Fig. 2:**
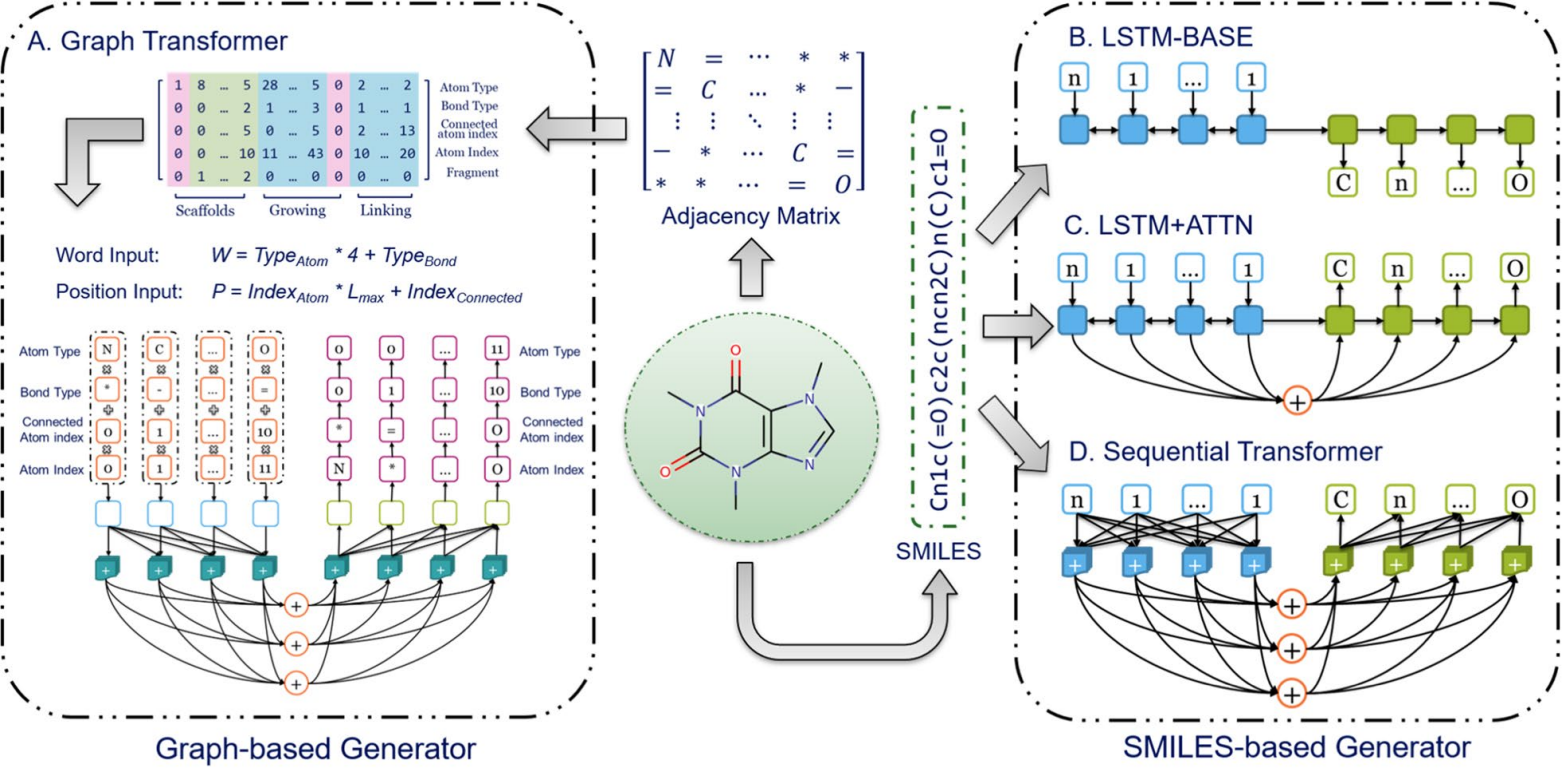
Architectures of four different end-to-end deep learning models: **A** The Graph Transformer; **B** The LSTM-based encoder-decoder model (LSTM-BASE); **C** The LSTM-based encoder-decoder model with attention mechanisms (LSTM + ATTN); **D** The sequential Transformer model. The Graph Transformer accepts a graph representation as input and SMILES sequences are taken as input for the other three models

Here, four different end-to-end DL architectures were compared to deal with different molecular representations of either graph or SMILES (Fig. 2). These methods included: (A) a Graph Transformer, (B) an LSTM-based encoder-decoder model (LSTM-BASE), (C) an LSTM-based encoder-decoder model with an attention mechanism (LSTM + ATTN) and (D) a Sequential Transformer model. All of these DL models were constructed with *PyTorch* [30].

For the SMILES-based models, three different types were constructed as follows (Fig. 2, right). The two LSTM-based models share similarities as will be outlined here. In the LSTM-BASE model (Fig. 2B) the encoder and decoder had the same architecture as used in *DrugEx v2*, containing one embedding layer, three recurrent layers, and one output layer. The number of neurons in the embedding and hidden layers were 128 and 512, respectively. The hidden states of the recurrent layer in the encoder are directly sent to the decoder as the initial states. The LSTM + ATTN modes is constructed on top of this LSTM-BASE model by adding an attention layer between the encoder and decoder (Fig. 2C). The attention layer calculates the weight for each position of the input sequence to determine which position the decoder needs to focus on during the decoding process. For each step the weighted sums of the output calculated by the encoder are combined with the output of the embedding layer in the decoder to form the input for the recurrent layers. The output of the recurrent layers is dealt with by the output layer to generate the probability distribution of tokens in the vocabulary.

The sequential Transformer has a distinct architecture compared to the LSTM-BASE and LSTM + ATTN models although it also exploits an attention mechanism. For the embedding layers “position encodings” are added into the typical embedding structure as the first layer of the encoder and decoder. This ensures that the model no longer needs to encode the input sequence token by token but can process all tokens in parallel. For the position embedding, sine and cosine functions are used to define its formula as follows:

$${PE}_{(p, 2i)}=\mathrm{sin}(pos/{10000}^{2i/{d}_{m}})$$

$${PE}_{(p, 2i+1)}=\mathrm{cos}(pos/{10000}^{2i/{d}_{m}})$$

where *PE(p, i)* is the *i*^*th*^ dimension of the position encoding at position *p*. It has the same dimension *d*_*m*_ = 512 as the typical embedding vectors so that the two can be summed.

In addition, self-attention is used in the hidden layers to cope with long-range dependencies. For each hidden layer in the encoder, it employs a residual connection around a multi-head self-attention sublayer and feed-forward sublayer followed by layer normalization. Furthermore, a third sublayer with multi-head attention is inserted to capture the information from output of the encoder.

This self-attention mechanism is defined as the scaled dot-product attention with three vectors: queries (*Q*), keys (*K*) and values (*V*), of which the dimensions are *d*_*q*_, *d*_*k*_,* d*_*v*_, respectively. The output matrix is computed as:

$$Attention\left(Q,K,V\right)=softmax\left(\frac{Q{K}^{\intercal }}{\sqrt{{d}_{k}}}\right)V$$

Instead of a single attention function, the Transformer adopts multi-head attention to combine information from different representations at different positions which is defined as:

$$\mathrm{MultiHead}\left(Q,K,V\right)=\mathrm{Concat}\left({head}_{1},\dots ,{head}_{h}\right){W}^{O}$$

where *h* is the number of heads. For each head, the attention values were calculated by different and learned linear projections with *Q*, *K* and *V* as follows*:*

$${head}_{i}=Attention(Q{W}_{i}^{Q}, K{W}_{i}^{K}, V{W}_{i}^{V})$$

where *W*^*O*^*, W*^*Q*^*, W*^*K*^ and* W*^*V*^ are metrics of learned weights and we set *h* = 8 as the number of heads and *d*_*k*_ = *d*_*v*_ = 64 in this work.

For the graph representation of the molecules, the structure of the sequential Transformer was updated to a Graph Transformer model (Fig. 2A). Similar to the sequential Transformer the Graph Transformer also requires the encoding of both word and position as the input. For the input word, the atom and bond cannot be processed simultaneously; therefore the indices of the atom and the bond are combined together as follows:

$$W={T}_{atom}\times 4+ {T}_{bond}$$

The index of the input word (*W*) for calculating word vectors is obtained by multiplying the atom type (*T*_*atom*_) by four (the total number of bond types defined) and subsequently add the bond index (*T*_*bond*_). Similarly, the position of each step cannot be used to calculate the position encoding directly. Faced with more complex data structure than sequential data, Dosovitskiy et al*.* proposed a new positional encoding scheme to define the position for each patch in image data for image recognition [31]. Inspired by their work the position encoding at each step was defined as:

$$P={I}_{Atom}\times {L}_{max}+ {I}_{Connected}$$

The input position (*P*) for calculating the position encoding was obtained by multiplying the current atom index (*I*_*Atom*_) by the max length (*L*_*max*_) and then adding the index of the connected atom (*I*_*Connected*_), which was then processed with the same positional encoding method as with the sequential Transformer. For the decoder, the hidden vector from the Graph Transformer was taken as the starting point to be decoded by a GRU-based recurrent layer; and the probability of atom type, bond type, connected atom index, and current atom index was decoded one by one sequentially.

When graph-based molecules are generated, the chemical valence rule is checked in every step. Invalid values of atom and bond types will be masked and an incorrect previous or current position will be removed  to ensure the validity of all generated molecules. It is worth noticing that before being encoded, each molecule will be kekulized, meaning that the aromatic rings will be transformed into either single or double bonds. The reason for this is that aromatic bonds interfere with the calculation of the valence value for each atom.

During the training process of SMILES-based models, a negative log likelihood function was used to construct the loss function. The loss function guarantees that the probability of the token at each step in the output sequence becomes large enough in the probability distribution of the vocabulary calculated by the deep learning model. In comparison, the loss function used by the Graph Transformer model also contains four parts for atom type, bond type, connected atom index and current atom index. Here the sum of these negative log probability values is minimized to optimize the parameters in the model. For this, the Adam algorithm was used for the optimization of the loss function. Here, the learning rate was set as 10^–4^, the batch size was 256, and training steps were set to 20 epochs for pre-training and 1,000 epochs for fine-tuning. In the end, optimal models were selected from the epoch in which the loss function achieved a minimum on the validation set. In the fine-tuning process, early stopping was evoked if the loss value did not decrease after 100 epochs.

### Multi-objective optimization

In order to combine multiple objectives, we exploited a Pareto-based ranking algorithm with GPU acceleration as mentioned in *DrugEx v2 *[25]. Given two solutions *m*_*1*_ and *m*_*2*_ with their scores (*x*_*1*_*, x*_*2*_*, …, x*_*n*_) and (*y*_*1*_*, y*_*2*_*, …, y*_*n*_), then *m*_*1*_ is said to Pareto dominate *m*_*2*_ if and only if:

$$\forall \mathrm{ j}\in \left\{1, \dots ,\mathrm{ n}\right\}: {x}_{j} \ge {y}_{j} \;and\; \exists \mathrm{j}\in \left\{1, \dots ,\mathrm{ n}\right\}: {x}_{j};{y}_{j}$$

Otherwise, neither *m*_*1*_ nor *m*_*2*_ is dominates. After the dominance between all pairs of solutions has been determined, the non-dominated scoring algorithm is exploited to obtain a rank of Pareto frontiers which consist of a set of solutions. In the same frontier, molecules were ranked based on the average Tanimoto-distance to other molecules instead of the commonly used crowding distance in the same frontier. Subsequently molecules with smaller average distances were ranked on the top. The final reward R^*^ is defined as:

$${R}^{*}=\left\{\begin{array}{c} 0.5+\frac{k-{N}_{undesired}}{{2N}_{desired}},\quad if\, desired\\ \frac{k}{{2N}_{undesired}}, \quad if\, undesired\end{array}\right.$$

here *k* is the index of the solution in the Pareto rank. Rewards of undesired and desired solutions will be evenly distributed in (0, 0.5] and (0.5, 0.1], respectively.

In this work, two objectives were considered: (1) the QED score [32] as implemented by RDKit (from 0 to 1) to evaluate the drug-likeness of each molecule (a larger value means more drug-like); (2) an affinity score towards the A_2A_AR which was implemented by a random forest regression model with Scikit-Learn [33]. The input descriptors consisted of 2048D ECFP6 fingerprints and 19D physico-chemical descriptors (PhysChem). PhysChem included: molecular weight, logP, number of H bond acceptors and donors, number of rotatable bonds, number of amide bonds, number of bridge head atoms, number of hetero atoms, number of spiro atoms, number of heavy atoms, the fraction of SP3 hybridized carbon atoms, number of aliphatic rings, number of saturated rings, number of total rings, number of aromatic rings, number of heterocycles, number of valence electrons, polar surface area, and Wildman-Crippen MR value. Again, it was determined if generated molecules are desired based on the Affinity score (larger than the threshold = 6.5). In addition, the SA score [34] was also exploited an independent measurement to evaluate the synthesizability of generated molecules, which is also calculated by RDKit.

### Reinforcement learning

A reinforcement learning framework was constructed based on the interplay between a Graph Transformer (agent) and two scoring functions (environment). A policy gradient method was implemented to train the reinforcement learning model, the objective function is designated as follows:

$$J\left(\theta \right)={\mathbb{E}}\left[{{R}^{*}(y}_{1:T})|\theta \right]=\sum_{t=1}^{T}logG\left({y}_{t}|{y}_{1:t-1}\right)\bullet {R}^{*}\left({y}_{1:T}\right)$$

For each step *t* during the generation process the generator (*G*) determines the probability of each token (*y*_*t*_) from the vocabulary based on the generated sequence in previous steps (*y*_*1:t-1*_). In the sequence-based models *y*_*t*_  is only a token selected from the vocabulary to construct SMILES while in the graph-based model it can be different type of atoms or bonds or the atoms connected by the bond. The parameters in the objective function are updated by a policy gradient based on the expected end reward (R^*^) received from the predictors. By maximizing this function, the parameter $\theta$ in the generator can be optimized to ensure that the generator designs desired molecules which obtain a high reward score.

In order to improve the diversity and reliability of generated molecules, the exploration strategy for molecule generation during the training loops was implemented. In the training loop the generator is trained to produce a chemical space as defined by the target of interest. In this strategy there are two networks with the same architectures, an exploitation net (*G*_*θ*_) and an exploration net (*G*_*φ*_). *G*_*φ*_ did not require training as the parameters are always fixed and it is based on the general drug-like chemical space for diverse targets obtained from ChEMBL. The parameters in *G*_*θ*_ on the other hand were updated for each epoch based on the policy gradient. Again, an *exploring rate* (*ε*) was defined with a range of [0.0, 1.0] to determine the percentage of scaffolds being randomly selected as input by *G*_*φ*_ to generate molecules*.* Conversely *G*_*θ*_ generated molecules with other input scaffolds. After the training process was finished *G*_*φ*_ was removed and only *G*_*θ*_ was left as the final model for molecule generation.

### Performance evaluation

In order to evaluate the performance of the generators, five coefficients were calculated from the population of generated molecules (validity, accuracy, desirability, uniqueness, and novelty) which are defined as:

$$Validity=\frac{{N}_{valid}}{{N}_{total}}$$

$$Accuracy=\frac{{N}_{accurate}}{{N}_{total}}$$

$$Desirability=\frac{{N}_{desired}}{{N}_{total}}$$

$$Uniqueness=\frac{{N}_{unique}}{{N}_{total}}$$

$$Novelty=\frac{{N}_{novel}}{{N}_{total}}$$

here *N*_*total*_ is the total number of molecules, *N*_*valid*_ is the number of molecules parsed as valid SMILES sequences, *N*_*accurate*_ is the number of molecules that contained all given scaffolds, *N*_*desired*_ is the number of desired molecules that reach all required objectives, *N*_*unique*_ is the number of molecules which are different from others in the dataset, *N*_*novel*_ is the number of generated unique molecules that do not exist in the *ChEMBL* set.

To measure molecular diversity, we adopted the Solow Polasky measurement as in the previous work. This approach was proposed by Solow and Polasky in 1994 to estimate the diversity of a biological population in an eco-system [35]. The formula to calculate diversity was redefined to normalize the range of values from [1, m] to (0, m] as follows:

$$I\left(A\right)=\frac{1}{\left|A\right|}{{\varvec{e}}}^{\intercal }{F({\varvec{s}})}^{-1}{\varvec{e}}$$

where *A* is a set of drug molecules with a size of *|A|* equal to *m*, ***e*** is an *m*-vector of 1’s and *F(s)* = [*f(d*_*ij*_*))*] is a non-singular *m* × *m* distance matrix. Hereing *f(d*_*ij*_*)* stands for the distance function of each pair of molecules provided as follows:

$$f\left(d\right)={e}^{-\theta {d}_{ij}}$$

Here θ was set to 0.5 as suggested in [35]. The distance *d*_*ij*_ between molecules *s*_*i*_ and *s*_*j*_ was defined by using the Tanimoto-distance with ECFP6 fingerprints as follows:

$${d}_{ij}=d\left({s}_{i}, {s}_{j}\right)=1-\frac{\left|{s}_{i}\cap {s}_{j}\right|}{\left|{s}_{i}\cup {s}_{j}\right|} ,$$

where |* s*_*i*_ ∩ *s*_*j*_ | represents the number of common fingerprint bits, and | *s*_*i*_ ∪ *s*_*j*_ | is the number of union fingerprint bits.

## Results and discussion

### Fragmentation of molecules

**Fig. 3:**
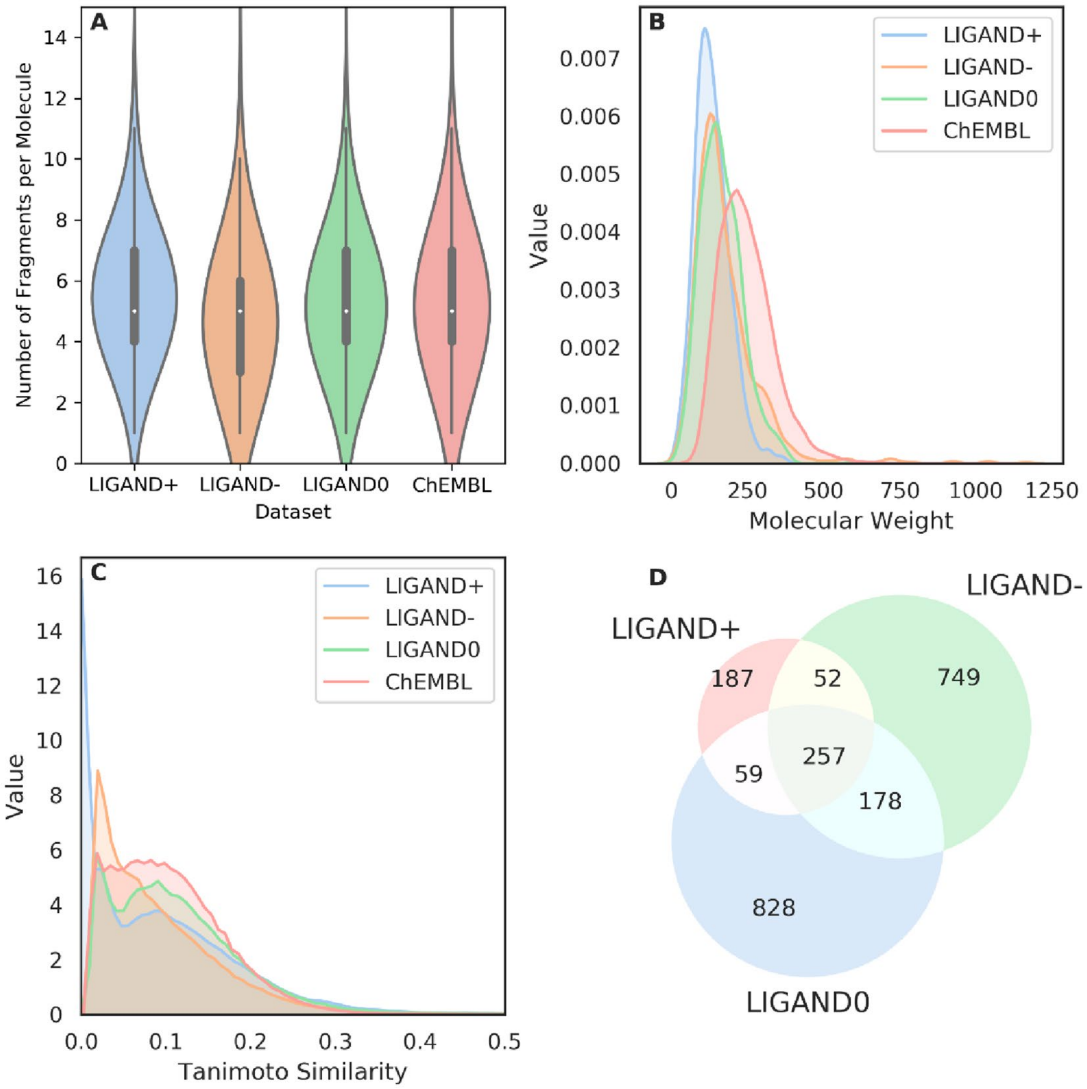
Analysis of some properties of fragments in the *ChEMBL* set and three *LIGAND* subsets. **A** Violin plot for the distribution of the number of fragments per molecules; **B** Distribution of molecular weight of these fragments; **C** Distribution of the similarity of the fragments measured by the Tanimoto-similarity with ECFP4 fingerprints; **D** Venn diagram for the intersection of the fragments existing in the three subsets of the *LIGAND* set

Each molecule was decomposed into a series of fragments with the BRICS algorithm to construct a fragment-molecule pair with a compiled elaborate set of rules. For the *ChEMBL* and *LIGAND* sets, 194,782 and 2,310  fragments were obtained, respectively. The LIGAND set was further split into three parts: active ligands (*LIGAND*^+^, 2,638 compounds), inactive ligands (*LIGAND*^−^, 2,710 compounds) and undetermined ligands (*LIGAND*^*0*^, 5,480 compounds) based on the pX of bioactivity for A_2A_AR. The number of fragments per each molecule in these four datasets have a similar distribution (Fig. 3A) and there are approximately five fragments on average for each molecule with a 95% confidence between [1, 11] (Fig. 3A).

In the *LIGAND* set the three subsets have a similar molecular weight distribution of the fragments (Fig. 3B) with an average of 164.3 Da, smaller than in the *ChEMBL* set (247.3 Da). The average similarity between training and test set is also slightly higher in the *LIGAND* set compared to the *ChEMBL* set (Additional file 1: Figure S1). As the structure of fragments is generally smaller than the structure of molecules we used the Tanimoto similarity calculation with ECFP4 (rather than ECFP6) descriptors between each pair of fragments in the same dataset to check the similarity of these fragments. It was found that most of them were smaller than 0.5 indicating that they are dissimilar to each other (Fig. 3C). Especially, the fragments in the *LIGAND*^+^ set have the largest diversity. Moreover, the distribution of different fragments in these three subsets of the *LIGAND* set is shown in Fig. 3D. The molecules in these three subsets have their unique fragments and share some common substructures.

## Generated molecules

### Pre-training and Fine-tuning

**Fig. 4:**
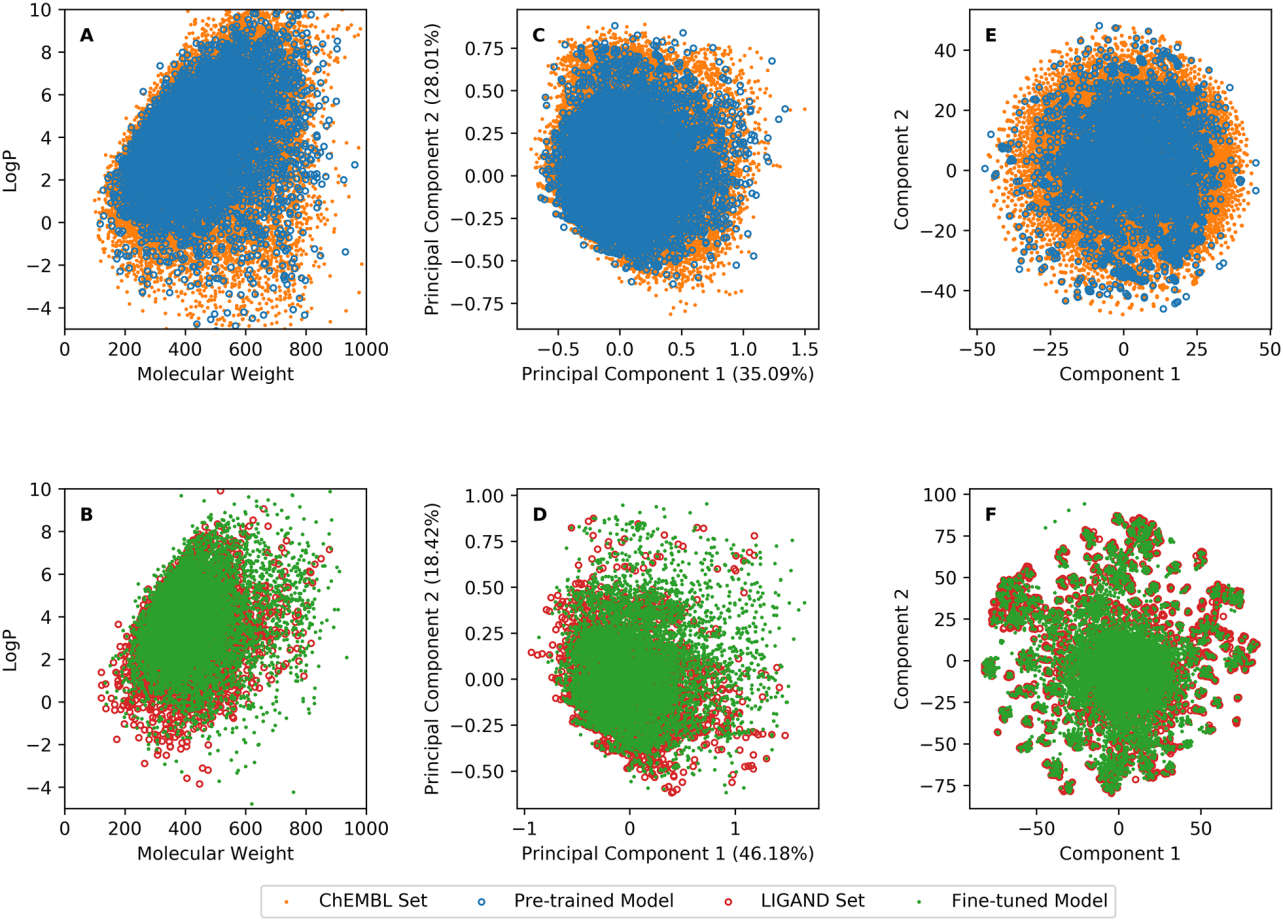
The chemical space of generated molecules by the Graph Transformer. Shown are the molecules generated by the models pre-training on the *ChEMBL* set (**A**, **C** and **E**) and fine-tuning on the *LIGAND* set (**B**, **D** and **F**). Chemical space was represented by either logP ~ MW (**A** and **B**) and the first two components from a PCA on PhysChem descriptors (**C**, **D**) and t-SNE on ECFP6 fingerprints (**E** and **F**)

After finishing the dataset construction four models were pre-trained on the *ChEMBL* set and fine-tuned on the *LIGAND* set. These models were benchmarked on a server with Nvidia Tesla P100 GPUs. After the training process converged, each fragment in the test set was presented as input 20 times for both *ChEMBL* and *LIGAND* test sets to generate molecules. The performance is shown in Table 1 (with additional tanimoto frequencies in the Additional file 1: Figure S2). Based on this benchmark, Transformer methods outperformed LSTM-based methods using SMILES. In addition, the training of Transformer models was found to be faster but to consume more computational resources than LSTM-based methods with the same number of neurons in the hidden layers. Although the three SMILES-based models improved after being fine-tuned they were still outperformed by the Graph Transformer because of the advantages of graph representation. To further check the accuracy of generated molecules the chemical space between the generated molecules and the compounds in the training set was compared with three different representations (1) MW ~ logP; (2) PCA with 19D PhysChem descriptors; (3) tSNE with 2048D ECFP6 fingerprints (Fig. 4). In addition, the loadings of PCA for each descriptor in PhysChem are provided in Additional file 1: Table S4. The region occupied by molecules generated by the Graph Transformer overlapped completely with the compounds in both the *ChEMBL* and *LIGAND* sets. In addition, the average tanimoto similarity of molecules generated by the four methods in pre-training, fine-tuning, and reinforcement learning using the Graph Transformer are shown in supplementary information (Additional file 1: Figure S3).

**Table 1:** The performance of four different generators with different number of neurons in hidden layers for pre-training and fine-tuning processes

Methods	Hidden Neurons	Pre-trained Model				Fine-tuned Model			
		Validity	Accuracy	Novelty	Uniqueness	Validity	Accuracy	Novelty	Uniqueness
Graph Transformer	512	100.0%	99.3%	99.9%	99.4%	100.0%	99.2%	68.9%	82.9%
Sequential Transformer	128	91.8%	62.4%	90.2%	92.5%	94.5%	80.5%	8.6%	24.3%
	256	94.2%	69.3%	89.3%	91.4%	98.8%	89.5%	9.2%	26.6%
	512	96.7%	74.0%	89.1%	91.8%	99.3%	92.7%	8.9%	28.9%
	1024	97.1%	77.9%	89.5%	91.4%	99.4%	94.3%	8.2%	32.9%
LSTM-BASE	128	87.1%	38.7%	83.2%	84.0%	85.2%	53.1%	9.9%	26.8%
	256	91.4%	48.8%	89.0%	91.2%	94.5%	75.8%	5.8%	21.2%
	512	93.9%	52.4%	84.3%	89.1%	98.7%	81.6%	3.9%	19.2%
	1024	95.7%	57.0%	79.6%	87.5%	99.6%	90.2%	2.1%	18.1%
LSTM + ATTN	128	89.8%	57.0%	84.2%	85.0%	85.2%	64.8%	14.2%	27.8%
	256	92.6%	68.4%	87.1%	89.5%	94.9%	80.5%	8.9%	22.4%
	512	94.3%	72.8%	85.3%	89.7&	96.9%	85.9%	6.3%	20.7%
	1024	96.0%	75.0%	80.7%	89.4%	99.1%	92.9%	4.2%	20.2%

The graph representation for molecules has advantages over the SMILES representation when dealing with fragment-based molecule design: (1) **Invariance on a local scale**: During the process of molecule generation, multiple fragments in a given scaffold can be put into any position in the output matrix without changing the order of atoms and bonds in that scaffold. (2) **Extendibility on a global scale**: When fragments in the scaffold are growing or being linked, they can be flexibly appended in the end column of the graph matrix while the original data structure does not need changing. (3) **Free of grammar**: Unlike in SMILES sequences there is no explicit grammar to constrain the generation of molecules, such as the parentheses for branches and the numbers for rings in SMILES; (4) **Accessibility of chemical rules**: For each added atom or bond the algorithm can detect if the valence of atoms is valid or not and mask invalid atoms or bonds in the vocabulary to guarantee the whole generated matrix can be successfully parsed into a molecule. Due to these four advantages the Graph Transformer generates molecules faster while using less computational resources.

**Fig. 5:**
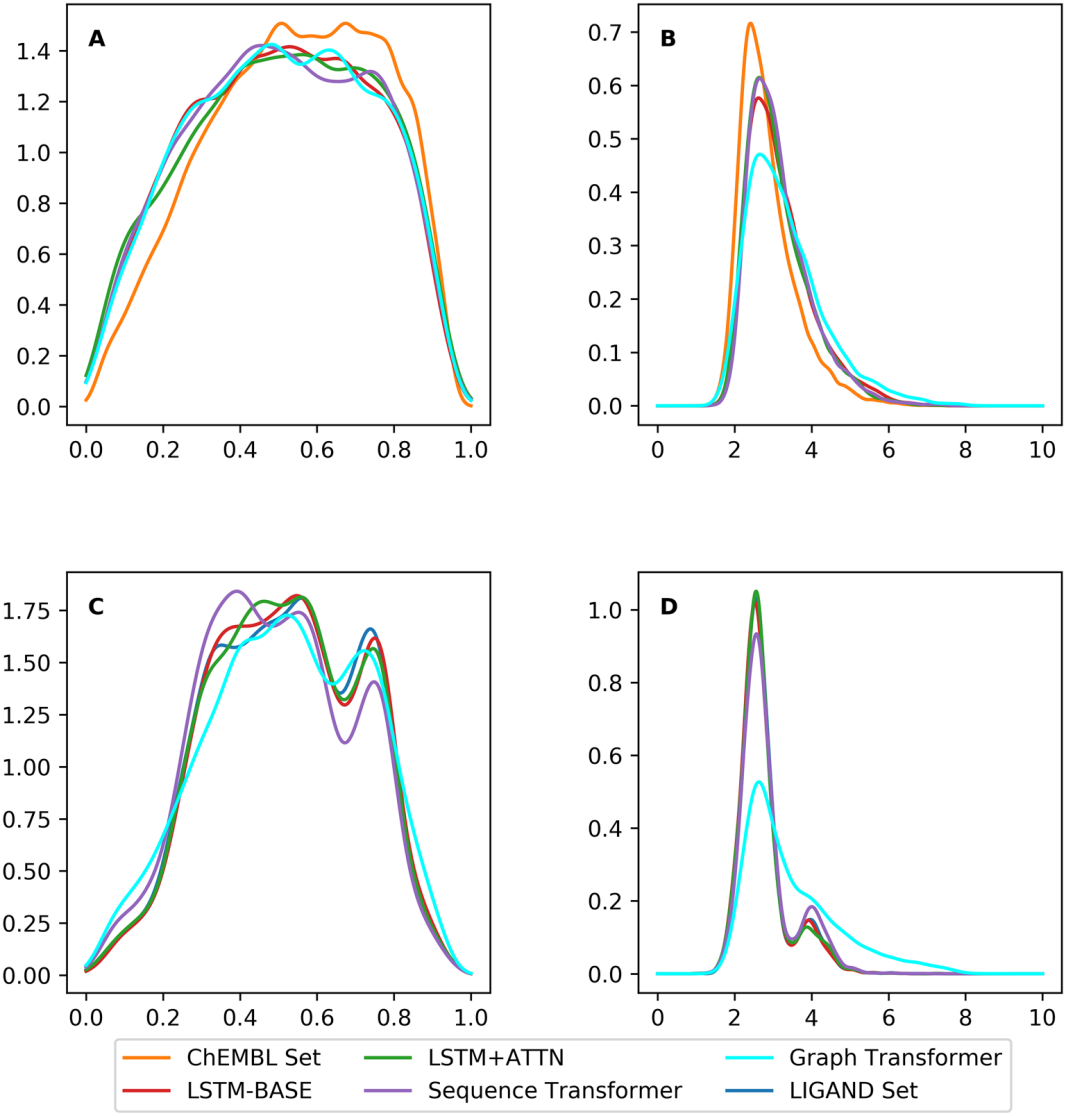
The distribution of the QED score (**A**, **C**) and SA score (**B**, **D**) of desired ligands. Shown are the molecules generated from the *ChEMBL* set and *LIGAND* set and of molecules generated by four different generators. For the QED score, four generators had the same performance as the molecules in both *ChEMBL* set (**A**) and the *LIGAND* set (**C**). For the SA score, Graph Transformer did not outperform three other SMILES-based generators in the *ChEMBL* set (**B**) and even worse in the *LIGAND* set (**D**).

However, after examining the QED scores and SA scores it was found that although the distribution of QED scores was similar between the methods (Fig. 5A and C), the synthesizability of the molecules generated by the Graph Transformer was not better than the SMILES-based generators. This was especially true when fine-tuning on the *LIGAND* set. A possible reason is that molecules generated by the Graph Transformer contain uncommon rings when the model dealt with long-distance dependencies. In addition, because of more complicated data structure and presence of more parameters in the model, the Graph Transformer did not outperform the other methods based on synthesizability of generated molecules while trained on a small dataset (*e.g.,* the *LIGAND* set). It is also worth noticing that there still was a small fraction of generated molecules that did not contain the required scaffolds which is caused by a kekulization problem. For example, a scaffold ‘CCC’ can be grown into ‘C1 = C(C)C = CC = C1’. After being sanitized, it can be transformed into ‘c1c(C)cccc1’. In this process one single bond in the scaffold is changed to an aromatic bond, which causes a mismatch between the scaffold and the molecule. Currently *DrugEx v3* cannot solve this problem because if the aromatic bond is taken into consideration, the valence of aromatic atoms is difficult to calculate accurately. This would lead to the generation of invalid molecules. Therefore, there is no aromatic bond provided in the vocabulary and all of the aromatic rings are inferred automatically through the molecule sanitization method in RDKit.

### Policy gradient

Because the Graph Transformer generates with the best performance it was chosen as the agent in the RL framework. Two objectives were tested in the training process. The first one was affinity towards A_2A_AR, which is predicted by the random forest-based regression model from *DrugEx v2*; the second one was the QED score calculated with RDKit to measure how drug-like a generated molecule is. With the policy gradient method as the reinforcement learning framework two cases were tested. On the one hand, predicted affinity for A_2A_AR was considered without the QED score. On the other hand, both objectives were used to optimize the model with Pareto ranking. After the training process finished, each fragment in the *LIGAND* test set was presented as input 20 times to generate molecules. In the first case 86.1% of the generated molecules were predicted active, while the percentage of predicted active molecules in the second case was 74.6%. Although the generator generated more active ligands without the QED score constraint, most of them are not drug-like as they frequently have a molecular weight larger than 500 Da. However, when we checked the chemical space represented by tSNE with ECFP6 fingerprints the overlap region between generated molecules and ligands in the training set was not complete implying that they fell out of the applicability domain of the regression model.

Changes to the exploration rate do not influence accuracy and have a low effect on diversity. However, desirability (finding active ligands) and uniqueness can be influenced significantly. Empirically determining an optimal value for a given chemical space is recommended.

In *DrugEx v2,* an exploration strategy simulated the idea of evolutionary algorithms such as *crossover* and *mutation* manipulations. However, when coupled to the Graph Transformer there were some difficulties and the strategy had to be given up. Firstly, the mutation strategy did not improve with different mutation rates. A possible reason is that before being generated, part the molecule was fixed with a given scaffold counteracting the effect of mutation caused by the mutation net. Secondly, the *crossover* strategy is computationally expensive in this context. This strategy needs the convergence of model training and iteratively updates the parameters in the agent. With multiple iterations, it takes a long period of time beyond the computational resources available. As a result, the exploration strategy was updated as mentioned in the Methods section with six different exploration rates: [0.0, 0.1, 0.2, 0.3, 0.4, 0.5].

**Fig. 6:**
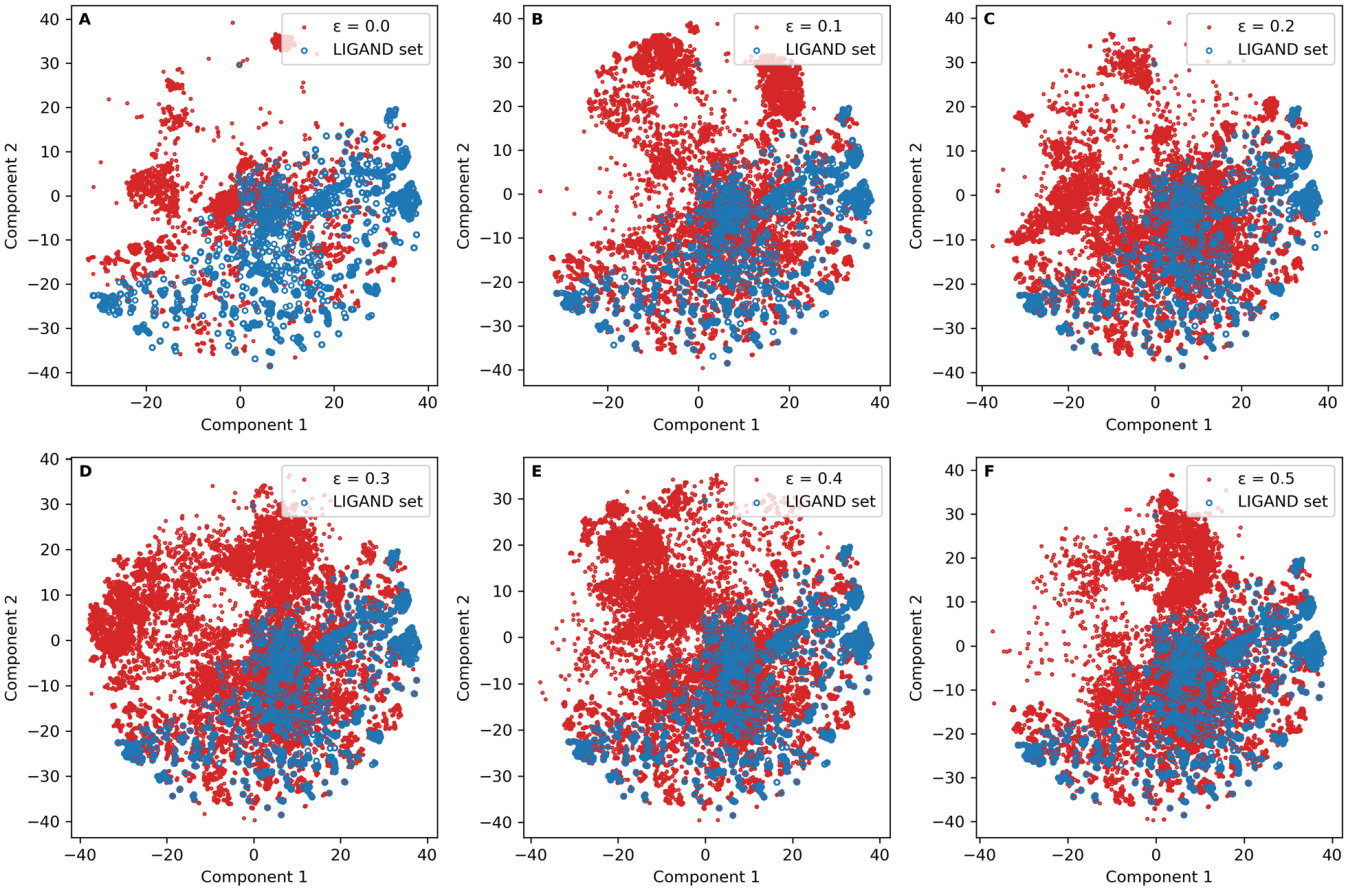
The chemical space of generated molecules by the Graph Transformer trained with different exploration rates in the RL framework. The chemical space was represented by t-SNE on ECFP6 fingerprints

After training the models, multiple scaffolds were input 20 times to generate molecules. The results for accuracy, desirability, uniqueness, novelty, and diversity with different exploration rates are shown in Table 2. With a low ε the model generated more desired molecules, but the uniqueness of the generated molecules decreased significantly. At ε = 0.3 the model generated the highest percentage of unique desired molecules (56.8%). Diversity was always larger than 0.84 and the model achieved the largest value (0.88) with ε = 0.0 or ε = 0.2. The chemical space represented by tSNE with ECFP6 fingerprints confirmed that the exploration strategy produced a set of generated molecules completely covering the region occupied by the *LIGAND* set (Fig. 6).

**Table 2:** The performance of the Graph Transformer with different exploration rates in the RL framework

ε	Accuracy	Desirability	Uniqueness	Novelty	Diversity
0.0	99.7%	74.6%	60.7%	60.6%	0.879
0.1	99.7%	66.8%	75.0%	74.6%	0.842
0.2	99.8%	61.6%	80.2%	79.4%	0.879
0.3	99.7%	56.8%	89.8%	88.8%	0.874
0.4	99.7%	54.8%	88.8%	87.5%	0.859
0.5	99.7%	46.8%	88.5%	86.4%	0.875

**Fig. 7:**
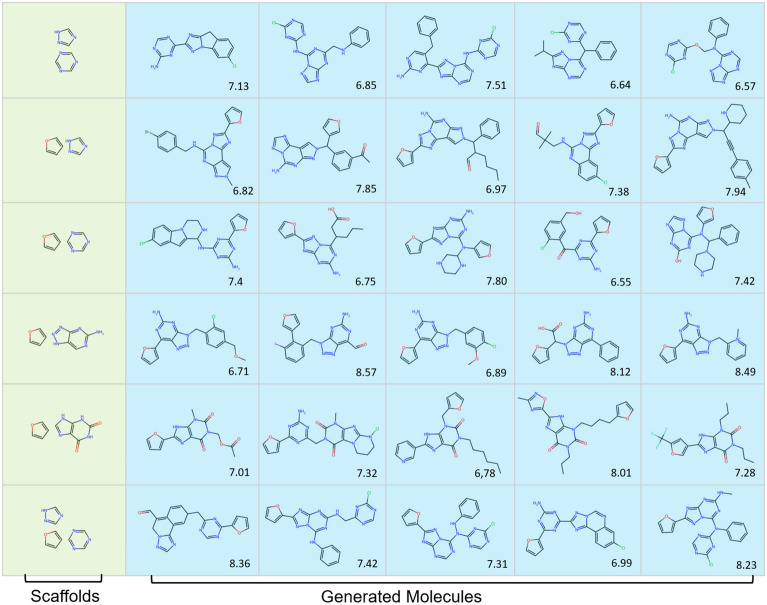
Sample of molecules generated with the Graph Transformer with different scaffolds. These scaffolds include: furan, triazine, aminotriazole, xanthine, and azapurine. The generated molecules based on the same scaffolds are aligned in the same row. The predicted pChEMBL value for the molecule is shown in the bottom right

In the chemical space making up antagonists of A_2A_AR there are several well-known scaffolds. Examples include furan, triazine, aminotriazole, and purine derivatives such as xanthine and azapurine. The Graph Transformer model produced active ligands for A_2A_AR (inferred from the predictors) with different combinations of these fragments as scaffolds. Taking these molecules generated by the Graph Transformer as an example, we filtered out the molecules with potentially reactive groups (such as aldehydes) and uncommon ring systems and listed 30 desired molecules as putative A_2A_AR ligands/antagonists (Fig. 7). For each scaffold five molecules were selected and assigned in the same row. These molecules are considered a valid starting point for further considerations and work (*e.g.,* molecular docking or simulation, or even synthesis).

## Conclusions and future perspectives

In this study, *DrugEx* was updated with the ability to design novel molecules based on scaffolds consisting of multiple fragments as input. In this version (*v3*), a new positional encoding scheme for atoms and bonds was proposed to make the Transformer model deal with a molecular graph representation. With one model, multiple fragments in a given scaffold can be grown at the same time and connected to generate a new molecule. In addition, chemical rules on valence are enforced at each step of the process of molecule generation to ensure that all generated molecules are valid. These advantages are impossible to be embodied in SMILES-based generation, as SMILES-based molecules are constrained by grammar that allows a 2D topology to be represented in a sequential way. With multi-objective reinforcement learning the model generates drug-like ligands, in our case for the A_2A_AR target.

In future work, the Graph Transformer will be extended to include other information as input to design drugs conditionally. For example, proteochemometric modelling (PCM) can take information for both ligands and targets as input to predict the affinity of their interactions, which allows generation of compounds that are promiscuous (useful for *e.g.,* viral mutants) or selective (useful for *e.g.*, kinase inhibitors) [36]. The Transformer can then be used to construct inverse PCM models which take the protein information as input (*e.g.,* sequences, structures, or descriptors) to design active ligands for a given protein target without known ligands. Moreover, the Transformer can also be used for lead optimization. For instance, the input can be a “hit” already, generating “optimized” ligands, or a “lead” with side effects to produce ligands with a better ADME/tox profile.

## Supplementary Information

**Additional file 1: Table S1.** Atoms in vocabulary for graph-based molecule generation. **Table S2.** The pseudo code for encoding the graph representation of molecules in DrugEx v3. **Table S3.** The pseudo code for decoding the graph representation of molecules in DrugEx v3. **Table S4.** Loadings of the PCA results of PCA on PhysChem descriptors between the molecules generated by pre-trained and fine-tuned Graph Transformer and the ChEMBL set and the LIGAND set, respectively. **Figure S1.** The distribution of Tanimoto similarity within training and test set, and between the sets for both the ChEMBL set (A) and the LIGAND set (B). **Figure S2.** The distribution of frequency of generated molecules based on the same fragments as input. These ligands were generated from pre-training (A) and fine-tuning (B) process. The molecules generated by the Graph Transformer model in the reinforcement learning process (C) were also counted the frequency of the same molecules for the same input fragments. **Figure S3.** The distribution of Tanimoto similarity between generated ligands and the molecules in the training set. These ligands were generated from pre-training (A) and fine-tuning (B) with four different models. The similarity was compared with the molecules in the ChEMBL, LIGAND sets, respectively. In addition, the ligands generated by the Graph Transformer model in the reinforcement learning (C) process with different hyperparameter ε were also compared the similarity with the LIGAND set.

## Data Availability

The data used in this study is publicly available ChEMBL data, the algorithm published in this manuscript is made available at https://github.com/CDDLeiden/DrugEx.
