# Supplementary material for: DrugEx v3: scaffold-constrained drug design with graph transformer-based reinforcement learning
Source: J Cheminform. 2023 Feb 20;15:24. doi: 10.1186/s13321-023-00694-z (PMC9940339; doi:10.1186/s13321-023-00694-z)
Supplement: Supplementary file 1 — Additional file 1: Table S1. Atoms in vocabulary for graph-based molecule generation. Table S2. The pseudo code for encoding the graph representation of molecules in DrugEx v3. Table S3. The pseudo code for decoding the graph representation of molecules in DrugEx v3. Table S4. Loadings of the PCA results of PCA on PhysChem descriptors between the molecules generated by pre-trained and fine-tuned Graph Transformer and the ChEMBL set and the LIGAND set, respectively. Figure S1. The distribution of Tanimoto similarity within training and test set, and between the sets for both the ChEMBL set (A) and the LIGAND set (B). Figure S2. The distribution of frequency of generated molecules based on the same fragments as input. These ligands were generated from pre-training (A) and fine-tuning (B) process. The molecules generated by the Graph Transformer model in the reinforcement learning process (C) were also counted the frequency of the same molecules for the same input fragments. Figure S3. The distribution of Tanimoto similarity between generated ligands and the molecules in the training set. These ligands were generated from pre-training (A) and fine-tuning (B) with four different models. The similarity was compared with the molecules in the ChEMBL, LIGAND sets, respectively. In addition, the ligands generated by the Graph Transformer model in the reinforcement learning (C) process with different hyperparameter ε were also compared the similarity with the LIGAND set. [file 13321_2023_694_MOESM1_ESM.docx]

**SUPPLEMENTARY INFORMATION**

**DrugEx v3: Scaffold-Constrained Drug Design with Graph Transformer-based Reinforcement Learning**

Xuhan Liu^1^, Kai Ye^2^, Herman W. T. van Vlijmen^1,3^, Adriaan P. IJzerman^1^, Gerard J. P. van Westen^1, *^

^1^Drug Discovery and Safety, Leiden Academic Centre for Drug Research, Einsteinweg 55, Leiden, The Netherlands

^2^School of Electrics and Information Engineering, Xi’an Jiaotong University, 28 XianningW Rd, Xi’an, China

^3^Janssen Pharmaceutica NV, Turnhoutseweg 30, B-2340, Beerse, Belgium

**^*^**To whom correspondence should be addressed: Gerard J. P. van Westen, Drug Discovery and Safety, Leiden Academic Centre for Drug Research, Einsteinweg 55, Leiden, The Netherlands. Tel: +31-71-527-3511. Email: [gerard@lacdr.leidenuniv.nl](mailto:gerard@lacdr.leidenuniv.nl).

Email Address of other authors: (1) Xuhan Liu: [x.liu@lacdr.leidenuniv.nl](mailto:x.liu@lacdr.leidenuniv.nl); (2) Kai Ye: [kaiye@xjtu.edu.cn](mailto:kaiye@xjtu.edu.cn); (3) Herman W. T. van Vlijmen: [hvvlijme@its.jnj.com](mailto:hvvlijme@its.jnj.com); (4) Adriaan P. IJzerman: [ijzerman@lacdr.leidenuniv.nl](mailto:ijzerman@lacdr.leidenuniv.nl)

**Table S1: Atoms in vocabulary for graph-based molecule generation.** The column of “Symbol” is the symbol of the atom and its charge; the column of “Valence” is the value of valence of the state of each chemical element; the “Number” column stands for the index of each element in the periodic table, the last row is the unique word for each state of these elements, a combination of its valence and symbol.

| Symbol | Valence | Charge | Number | Word |
| --- | --- | --- | --- | --- |
| O | 2 | 0 | 8 | 2O |
| O+ | 3 | 1 | 8 | 3O+ |
| O- | 1 | -1 | 8 | 1O- |
| C | 4 | 0 | 6 | 4C |
| C+ | 3 | 1 | 6 | 3C+ |
| C- | 3 | -1 | 6 | 3C- |
| N | 3 | 0 | 7 | 3N |
| N+ | 4 | 1 | 7 | 4N+ |
| N- | 2 | -1 | 7 | 2N- |
| Cl | 1 | 0 | 17 | 1Cl |
| S | 2 | 0 | 16 | 2S |
| S | 6 | 0 | 16 | 6S |
| S | 4 | 0 | 16 | 4S |
| S+ | 3 | 1 | 16 | 3S+ |
| S+ | 5 | 1 | 16 | 5S+ |
| S- | 1 | -1 | 16 | 1S- |
| F | 1 | 0 | 9 | 1F |
| I | 1 | 0 | 53 | 1I |
| I | 5 | 0 | 53 | 5I |
| I+ | 2 | 1 | 53 | 2I+ |
| Br | 1 | 0 | 35 | 1Br |
| P | 5 | 0 | 15 | 5P |
| P | 3 | 0 | 15 | 3P |
| P+ | 4 | 1 | 15 | 4P+ |
| Se | 2 | 0 | 34 | 2Se |
| Se | 6 | 0 | 34 | 6Se |
| Se | 4 | 0 | 34 | 4Se |
| Se+ | 3 | 1 | 34 | 3Se+ |
| Si | 4 | 0 | 14 | 4Si |
| B | 3 | 0 | 5 | 3B |
| B- | 4 | -1 | 5 | 4B- |
| As | 5 | 0 | 33 | 5As |
| As | 3 | 0 | 33 | 3As |
| As+ | 4 | 1 | 33 | 4As+ |
| Te | 2 | 0 | 52 | 2Te |
| Te | 4 | 0 | 52 | 4Te |
| Te+ | 3 | 1 | 52 | 3Te+ |
| * | 0 | 0 | 0 | * |

**Table S2: The pseudo code for encoding the graph representation of molecules in** ***DrugEx v3***

| **Algorithm** encoding:  **Input**:  **scaffold**: the structure of scaffold containing a series of fragments; **molecule**: the structure of molecule containing all of the fragments.  **output**:  **graph**: n x 5 matrix to represent encoded graph for each molecule.  scaffolds, growths, linkers <- [], [], []  start ← [‘GO’, 0, 0, 0, 0]  end ← [‘EOS’, 0, 0, 0, 0]  **For** atom **in** scaffold:  **If** atom **is** the first **in** each fragment:  **Insert** [atom.symbol, 0, 0, 0, atom.frag_index] **into** scaffolds  neighbors ← frags.GetNeighbors(atom)  **For** neighbor **in** neighbors:  **If** neighbor.index < atom.index: **continue**  item ← BOND_INFO(atom, neighbor)  item[-1] ← INDEX (atom.fragment)  **If** neighbor.fragment **==** atom.fragment:  **Insert** item **into** scaffolds  **Else**:  **Insert** item **into** linkers  **End**  **End**  **For** atom **in** mol **and not in** frags:  neighbors ← mol.GetNeighbors(atom)  **For** neighbor in neighbors:  if neighbor.index < atom.index: continue  item ← **BOND_INFO**(atom, neighbor)  **End**  graph ← **CONCATENATE**(start, scaffolds, growths, end, linkers, end)  **Return** graph  **Function** BOND_INFO(atom, neighbor):  bond ← mol.GetBond(atom, neighbor)  **If** neighbor.index < atom.index: continue  **If** neighbor **is** first **in** neighbors:  Item ← [atom.symbol, bond.symbl, atom.index, neighbor.index, 0]  **Else:**  item ← [0, bond.symbol, atom.index, neighbor.index, 0]  **Return** item |
| --- |
|  |

**Table S3: The pseudo code for decoding the graph representation of molecules in** ***DrugEx v3***

| **Algorithm** decoding:  **Input:**  **graph**: the n x 5 matrix to represent each molecule into a graph  **Output:**  **molecule:** structure of the kekulized molecule  **scaffold:** structure of the scaffold containing multiple fragments  molecule ← **new** MOL ()  scaffold ← **new** SUB ()  **For** atom, bond, prev, curr, scf **in** matrix:  **If** atom == 'EOS' **or** atom == 'GO':  **continue**  **If** atom != '*':  a ← **new** Atom (atom)  SET_FORMAL_CHARGE (a)  ADD_ATOM (molecule, a)  **If** scf != 0:  ADD_ATOM (scaffold, a)  **If** bond != 0:  b ← **new** Bond (bond, prev, curr)  ADD_BOND (molecule, b)  **If** frag != 0:  ADD_BOND (scaffold, b)  **End**  # automatically determine the aromatic rings  molecule ← SANITIZE (molecule)  scaffold ← SANITIZE (scaffold)  **Return** mol, subs |
| --- |
|  |
|  |

**Table S4: Loadings of the PCA results of PCA on PhysChem descriptors between the molecules generated by pre-trained and fine-tuned Graph Transformer and the *ChEMBL* set and the *LIGAND* set, respectively.**

| Descriptor | *ChEMBL* set | | *LIGAND* set | |
| --- | --- | --- | --- | --- |
|  | **PC1** | **PC2** | **PC1** | **PC2** |
| Molecular weight | 115.51 | -5.47 | 105.66 | -6.08 |
| logP | 0.70 | -1.35 | 0.35 | -1.49 |
| Number of H bond acceptors | 1.85 | 1.70 | 2.02 | 2.05 |
| Number of H bond donors | 0.66 | 1.14 | 0.60 | 0.99 |
| Number of rotatable bonds | 2.34 | 0.51 | 2.08 | 0.15 |
| Number of amide bonds | 0.38 | 0.27 | 0.29 | 0.14 |
| Number of bridge head atoms | 0.12 | -0.05 | 0.18 | -0.04 |
| Number of hetero atoms | 2.17 | 1.40 | 2.23 | 1.58 |
| Number of spiro atoms | 8.07 | -0.36 | 7.28 | -0.16 |
| Number of heavy atoms | 0.02 | -0.01 | 0.01 | -0.01 |
| Fraction of SP3 hybridized carbon atoms | 0.03 | 0.00 | 0.05 | 0.02 |
| Number of total rings | 0.77 | -0.43 | 0.80 | -0.07 |
| Number of aliphatic rings | 0.40 | -0.19 | 0.50 | -0.07 |
| Number of aromatic rings | 0.37 | -0.24 | 0.30 | 0.00 |
| Number of saturated rings | 0.27 | -0.13 | 0.31 | 0.07 |
| Number of heterocycles | 0.30 | -0.05 | 0.51 | 0.38 |
| Polar surface area | 25.08 | 32.30 | 24.33 | 31.57 |
| Number of valence electrons | 43.34 | -0.91 | 39.23 | -0.05 |
| Wildman-Crippen MR value | 29.90 | -4.78 | 26.97 | -4.86 |


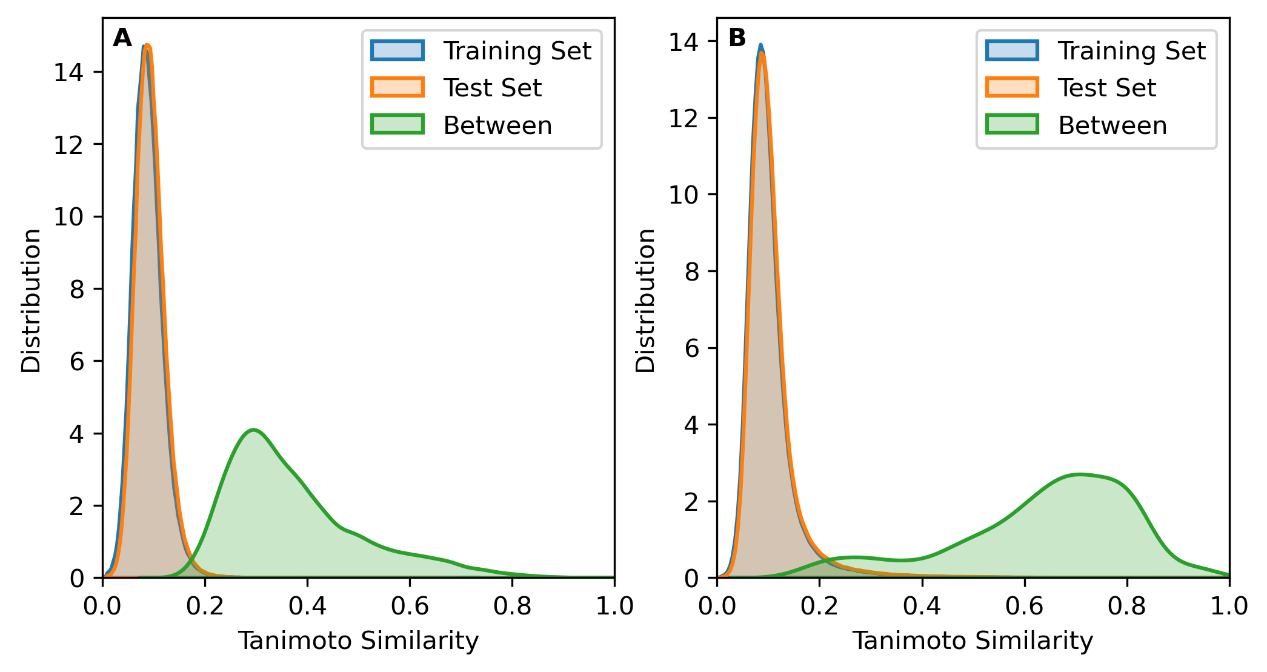


**Figure S1:** The distribution of Tanimoto similarity within training and test set, and between the sets for both the *ChEMBL* set (A) and the *LIGAND* set (B).


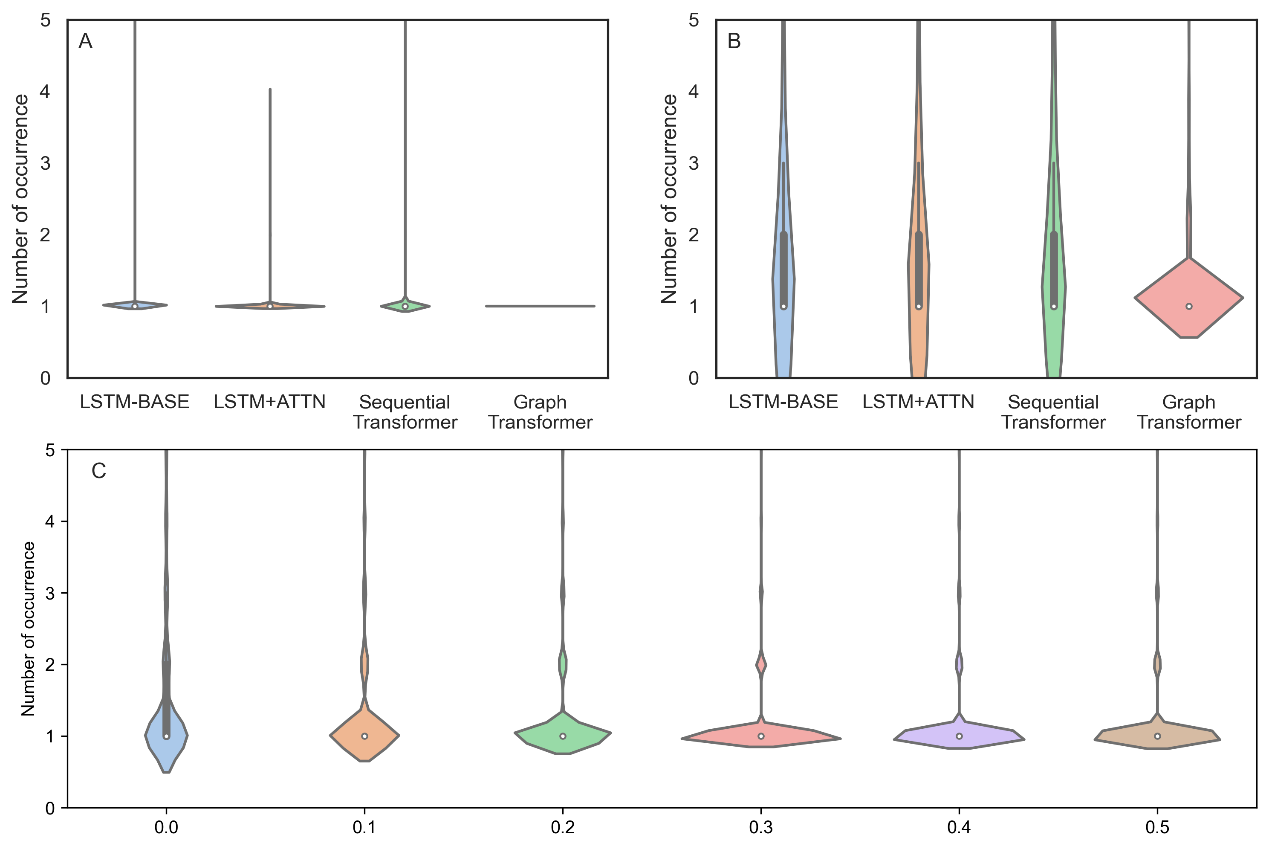


**Figure S2**: The distribution of frequency of generated molecules based on the same fragments as input. These ligands were generated from pre-training (A) and fine-tuning (B) process. The molecules generated by the Graph Transformer model in the reinforcement learning process (C) were also counted the frequency of the same molecules for the same input fragments.


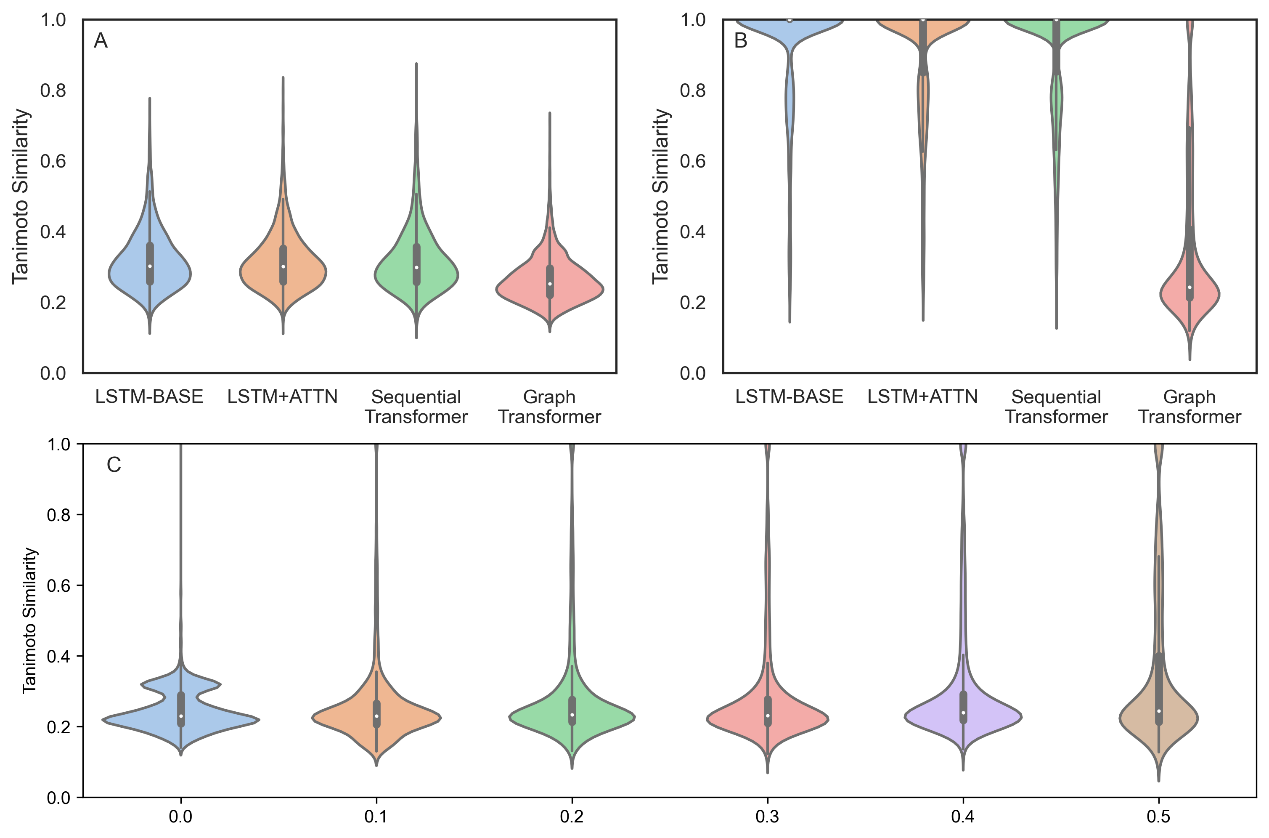


**Figure S3:** The distribution of Tanimoto similarity between generated ligands and the molecules in the training set. These ligands were generated from pre-training (A) and fine-tuning (B) with four different models. The similarity was compared with the molecules in the *ChEMBL*, *LIGAND* sets, respectively. In addition, the ligands generated by the Graph Transformer model in the reinforcement learning (C) process with different hyperparameter ε were also compared the similarity with the *LIGAND* set.
